# Photoluminescence and Stability of Dion–Jacobson Tin-Based Halide Perovskites with Different Spacer Cation Chain Length

**DOI:** 10.3390/molecules30030703

**Published:** 2025-02-05

**Authors:** Muhammad Umair Ali, Wen Ting Sun, Aleksandr A. Sergeev, Atta Ur Rehman, Kam Sing Wong, Aleksandra B. Djurišić, Jasminka Popović

**Affiliations:** 1 Department of Physics, The University of Hong Kong, Pokfulam Road, Hong Kong; umairalisabir@outlook.com (M.U.A.); wsunae@gmail.com (W.T.S.); geoatta@hku.hk (A.U.R.); 2 Department of Physics and William Mong Institute of Nano Science and Technology, The Hong Kong University of Science and Technology, Clearwater Bay, Hong Kong; sergeev@ust.hk (A.A.S.); phkswong@ust.hk (K.S.W.); 3 Ruđer Bošković Institute, Bijenička 54, 10000 Zagreb, Croatia

**Keywords:** tin-based halide perovskite, photoluminescence, white emission

## Abstract

Two-dimensional tin halide perovskites are of significant interest for light emitting applications. Here, we investigate the effect of organic cation A on the stability of different Dion–Jacobson tin-based halide perovskites. The ASnBr_4_ materials using diammonium cation A with shorter alkyl chains are found to exhibit improved stability, exhibiting dramatic stability difference between the most stable HDASnBr_4_, where HDA denotes 1,6-hexanediammonium, and two materials with 8- and 10-carbon alkyl chain ammonium cations. The HDASnBr_4_ powders were thermally stable at 100 °C in an argon environment but exhibited decreasing photoluminescence with time in ambient air at 100 °C. The sample degradation at 100 °C is accelerated compared to room temperature, but it proceeds along similar pathways, namely phase transformation followed by perovskite decomposition. Light emission from HDASnBr_4_ thin films could be further enhanced by methanol vapor treatment, and warm white emission with Commission Internationale de l’Eclairage (CIE) coordinates (0.37, 0.34) could be obtained by combining HDASnBr_4_ with a blue-emitting polymer film, while direct mixing of blue phosphor and HDASnBr_4_ powder yields white emission with CIE coordinates of (0.34, 0.32).

## 1. Introduction

Lead-free halide materials, in particular tin-based halide perovskites, have been attracting significant interest for light emitting applications in recent years [1,2,3,4,5,6,7,8,9,10,11,12,13,14,15,16,17,18,19,20,21,22,23,24,25,26,27]. While lead-based halide perovskites have achieved excellent progress in both photovoltaic and light emitting applications in terms of efficiency which has reached the comparable levels to more mature technologies, the toxicity of lead has resulted in interest in the development of less toxic, lead-free halide perovskite materials [1]. Among lead-free perovskites, tin-based materials are most commonly studied due to their similarity with Pb-containing counterparts [1]. However, Sn-based perovskites are generally less stable compared to Pb-based ones, as Sn^2+^ can be readily oxidized to Sn^4+^ [1]. For this reason, more stable forms of Sn-based perovskites, such as 2D tin-based perovskite materials, have been attracting increasing attention [1]. Two-dimensional tin-based perovskites can either exhibit a typical narrow band emission determined by the halide used, or they exhibit a broadband emission covering a large part of the visible spectrum, commonly attributed to self-trapped excitons (STE) [1,3,7,8]. STE emission, in general, is commonly observed in tin-based halide perovskites with different crystal structures, ranging from 0D perovskitoid structures to 2D perovskites [3,4,5,7,9,10,11,12,15,21,22,23,24,25,26,27]. Due to their broad emission spectrum, high photoluminescence quantum yield (PLQY), and low self-absorption, perovskites exhibiting STE emission are of significant interest for phosphor applications in light emitting devices [8]. In addition, since these materials do not contain rare earths, they offer an attractive low-cost alternative to current commercial phosphors which typically contain Eu, Ce, Tb, or Y rare earths [17].

A large number of different organic cations is capable of forming 2D perovskites, but the relationship between material properties and spacer cation structure is generally not well understood and comparative studies of materials with different spacer cations have been scarce. However, understanding of the relationship between spacer cation, light emitting properties and material stability is essential for practical applications. While several studies have reported exceptional ambient stability of different Dion–Jacobson (DJ) tin-based bromide perovskites [7,21,22,23,24] and there are comparisons of optical properties for different spacer cations for both Ruddlesden–Popper (RP) [10,19,27] and DJ tin halide perovskites [3], comparative studies of stability have been lacking. It is known that the spacer cation used can have a significant effect on the light emitting properties of the perovskite [3,10,27]. It has also been shown that 2D Sn-based perovskites which exhibit STE commonly consist of a dark stoichiometric 2D phase (A_2_SnBr_4_, where A is the spacer cation for an RP perovskite) and a bright phase with excess organic halide (A_2+x_SnBr_4+x_) [10]. For DJ perovskites, more complex stoichiometry differences have been reported for dark and bright phases in alcohol-doped samples [21]. Nevertheless, in all cases two different phases have widely different PLQYs [10], and the PLQY is also dependent on the spacer cation used [3,10]. Since the exposure to ambient air has been shown to affect the proportion of different phases in the DJ perovskite samples [7], we investigated the effect of the spacer cation choice on the sample stability. As DJ perovskites generally exhibit good stability when exposed to ambient at room temperature [7,24], we have investigated the stability at an elevated temperature (100 °C) to accelerate the degradation, as DJ perovskite samples typically exhibit faster decrease in photoluminescence (PL) intensity in ambient air at 100 °C compared to room temperature [21]. For the spacer cations, we have selected alkyl chains with different lengths (6, 8, and 10 carbon atoms) to ensure that we are considering spacer cations with similar chemical structure.

Thus, here we investigated the stability of HDASnBr_4_, ODASnBr_4_, and DDASnBr_4_, where HDA denotes 1,6-hexanediammonium, ODA denotes 1,8-octanediammonium, and DDA denotes 1,10-decanediammonium, using PL measurements for different times of exposure to ambient air. We have conducted comprehensive investigation of the crystal structure of the samples before and after exposure to air for different times. We found that HDASnBr_4_ not only exhibits the highest emission intensity in agreement with previous work [3], but it also exhibits the highest stability. Thus, HDASnBr_4_ thin film was used to demonstrate the achievement of warm white emission in combination with a blue-emitting polymer, achieving Commission Internationale de l’Eclairage (CIE) coordinates of (0.37, 0.34). In addition, white emission with CIE coordinates of (0.34, 0.32) was attained by direct mixing of blue phosphor and HDASnBr_4_ powder yields.

## 2. Results and Discussion

**Figure 1:**
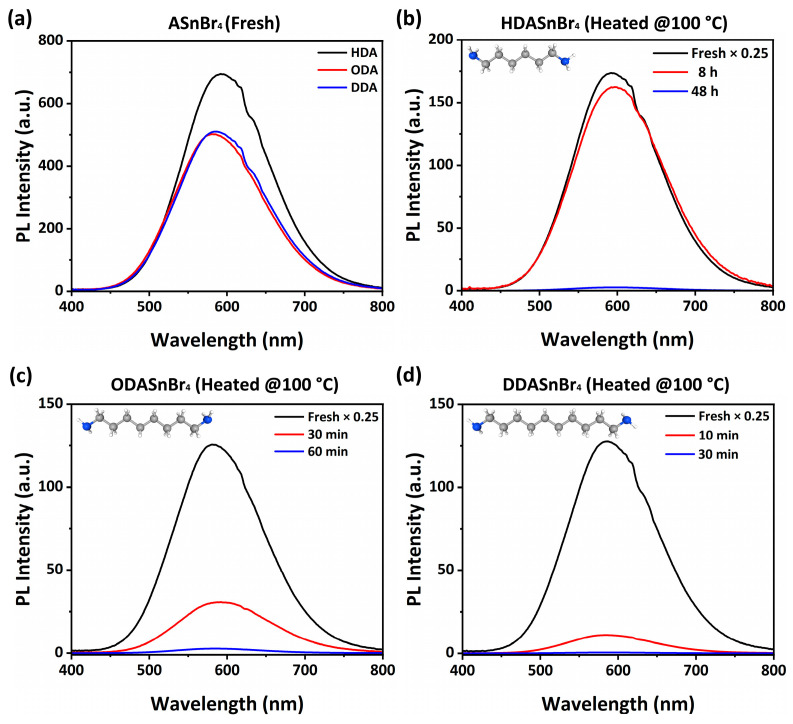
(**a**) Photoluminescence of ASnBr_4_ as a function of time in ambient air at 100 °C for (**b**) A = HDA, (**c**) A = ODA, and (**d**) A = DDA.

Figure 1 shows PL of ASnBr_4_ perovskite powders for different spacer cations. The PLQY trends of the samples follow the relationship ODA < DDA < HDA, as shown in Figures S1 and S2, but the absolute value of PLQY is dependent on the measurement conditions (see Figures S1c, S2c, and S3 for comparison of HDASnBr_4_ samples) due to experimental artifacts related to scattering and re-absorption, as discussed in Note S1. It should also be noted that there is a large variation in the PLQY values reported for this class of materials in the literature, ranging from low values [7,10,27] even as low as <1% [10] to >90% [3,10]. The differences in PLQY can potentially occur due to differences in measurement conditions (see Note S1), or they could be due to different spacer cation [3,27], a different proportion of existing crystal phases in the samples [7,10], exposure to protic solvents [21], and a likely synthesis method as well since the synthesis method would influence crystal structure, morphology, and solvent doping. The development of the correct measurement protocol for the determination of the PLQY of powder perovskite samples and a comparative investigation of different synthesis methods for the same spacer cation is of interest for future study, but here we focus on the investigation of the effect of spacer cation on stability using a synthesis method that typically produces a mixture of phases [7], as this allows us to investigate what is the effect of the phase composition of the samples on their stability.

As shown in Figure 1a, the strongest emission is obtained from HDA spacer which is in agreement with the literature report for a different synthesis method [3]. The samples have also been heated to 100 °C in ambient air (~55–65% RH) as shown in Figure 1b–d. Heating has been used to accelerate stability testing since these materials exhibit excellent long-term stability for storage at room temperature [7,24]. In addition, stability at elevated temperature is practically relevant for phosphor applications, as the phosphor temperature is expected to increase due to Joule heating of the light emitting diode. We can observe a dramatic stability difference for HDASnBr_4_ compared to ODASnBr_4_ and DDASnBr_4_; while the intensity of PL for ODA- and DDA-based materials diminishes after 60 and 30 min, respectively, the HDA-based material retains significant emission even after 8 h.

**Figure 2:**
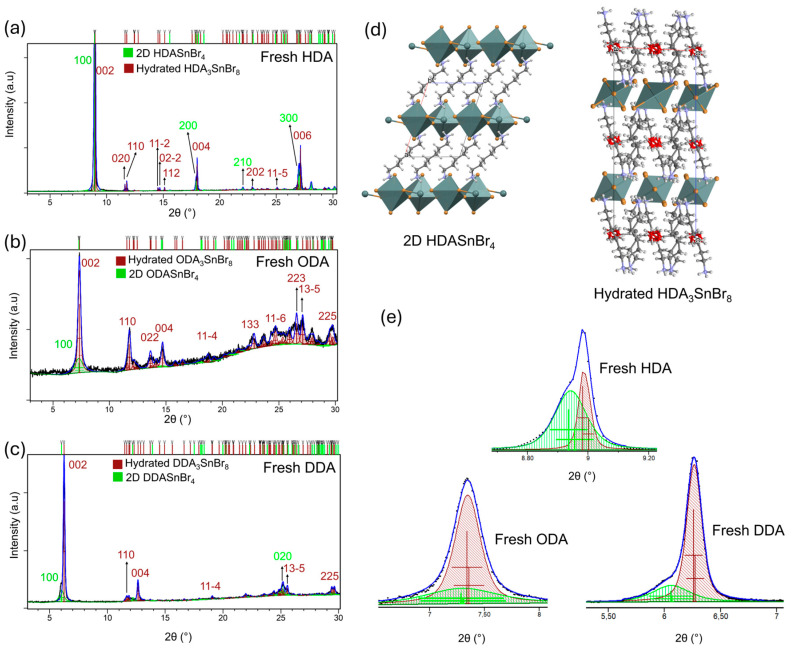
Le Bail refinement of (**a**) HDA-based perovskite, (**b**) ODA-based perovskite, (**c**) DDA-based perovskite. Experimental diffraction patterns are shown as black lines while the calculated patterns for are represented by blue lines. Diffraction lines corresponding to the 2D phase are marked with green vertical lines while the reflections of the 1D phase are marked with dark red vertical bars. (**d**) Structures of the 2D HDASnBr_4_ phase and 1D HDA_3_SnBr_8_·(H_2_O) phase, Sn-octahedra are given in teal, bromides are shown as orange balls while carbons, nitrogens, oxygens, hydrogens are gray, light blue, red, and white balls, respectively; (**e**) enlarged part of the XRD patterns showing the most prominent diffraction lines for as-prepared HDA, ODA, and DDA samples. Diffraction lines corresponding to the 2D phase are colored in green while the reflections of the 1D phase are colored in dark red.

Structural analysis of fresh, as-prepared HDA-, ODA- and DDA-based perovskites has been performed by the means of powder X-ray diffraction (XRD) as shown in Figure 2.

From Figure 2a, we can observe that the fresh HDA-based sample contained the 2D HDASnBr_4_ perovskite phase and 1D HDA_3_SnBr_8_·(H_2_O) phase. A previously published structure of HDAPbBr_4_ [28] was used for the refinement of HDASnBr_4_. The Le-Bail fitting showed that HDASnBr_4_ exhibits the longest-axis periodicity of 11.48 Å which is smaller than reported value for HDAPbBr_4_ (12.02 Å), as expected since Sn^2+^ is smaller than Pb^2+^. The structure as reported by Ning et al. [11] was used for the refinement of the 1D phase, HDA_3_SnBr_8_·(H_2_O). Similar to the case of HDA, Figure 2b,c show that as-prepared ODA and DDA samples also contain both 2D and hydrated 1D phases. The shift in most prominent diffraction lines (100 of 2D phase and 002 of 1D phase) towards lower 2θ angles can be observed which is expected considering the increase in the length of spacers; as the number of carbon atoms of spacers increases from HDA (6 atoms) to ODA (8 C atoms) to DDA (10 C atoms) the distance between the inorganic layers consequently also increases leading to the shift in the diffraction lines towards larger *d*-values (smaller 2θ angles). It is important to notice that although all three as-prepared samples contain both 2D and 1D phases, the amount of phases present in the samples are significantly different; from Figure 2e we can observe that the ODA and DDA samples dominantly contain 1D phase with a smaller amount of the 2D perovskite phase also present. On the other hand, the HDA sample contains approximately two times the quantity of 2D phase relative to 1D phase. Quantitative analysis of the fresh samples is given in Table S1.

There are different factors that can affect the stability of tin-based perovskites, and their degradation is commonly attributed to the oxidation of Sn^2+^ to Sn^4+^ [29,30,31], although recent reports indicate that the loss of organic cation rather than an increase in Sn^4+^ is responsible for the degradation [32]. The tin oxidation process generally readily occurs and it can be easily exacerbated by oxidizing solvent such as dimethylsulfoxide (DMSO) [29]. Consequently, it has been extensively studied in 3D perovskites for solar cell applications and it is usually addressed by reducing agents [30]. However, it has also been shown that the use of HDA can improve the stability of Sn-based perovskite solar cells and retard the oxidation of Sn^2+^ to Sn^4+^ [31], which indicates that DJ perovskites could be less susceptible to Sn^2+^ oxidation. In addition, while it was shown that the proportion of oxidized Sn^4+^ is lower in the bright phase (solvent-doped) compared to the dark (pristine) phase for ODASnBr_4_ [23], the Sn^4+^ content as a function of time of ambient air exposure was not experimentally investigated, and the improved stability with alcohol doping was simply attributed to the resistance to oxidation [21]. However, we have previously shown that there is no clear relationship between the normalized Sn^2+^/Sn^4+^ ratio and the time of ambient exposure for HDASnBr_4_ [7]. While some effect of Sn^2+^ oxidation on stability is expected, and indeed evident from reported faster degradation of samples prepared with a higher proportion of DMSO^7^, the fact that normalized Sn^2+^/Sn^4+^ does not simply decrease over time as PL decreases indicates that there are additional factors which have more significant influence on PL degradation. As we have previously shown that phase transformations significantly affect the emission intensity of HDASnBr_4_ for different times of ambient air exposure [7], we have therefore focused on analyzing the sample structure after heating in ambient.

**Figure 3:**
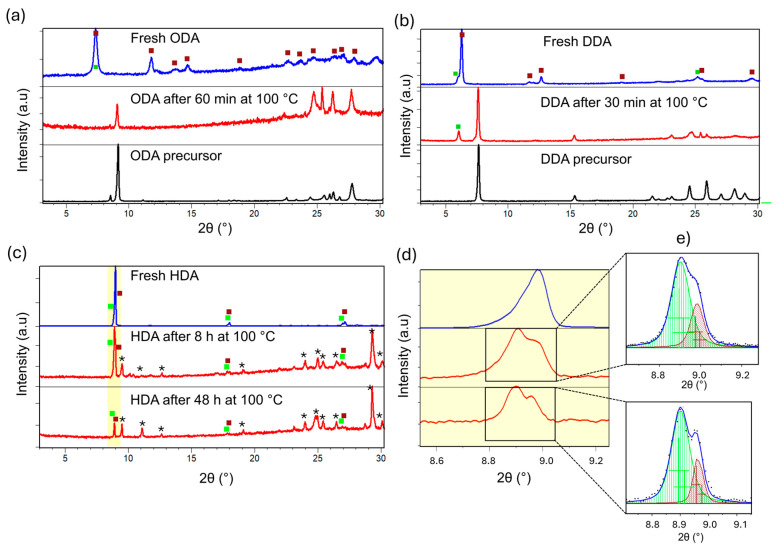
XRD patterns of thermally treated samples with different spacers: (**a**) ODA, (**b**) DDA, (**c**) HDA; unidentified phase is denoted by *, (**d**) enlarged part of XRD pattern for HDA sample, (**e**) Le Bail refinement of diffraction lines at ~8.9°2θ. The diffraction line corresponding to the 2D phase is colored in green while the reflection of the 1D phase is colored in dark red.

Thus, XRD analysis has been conducted after thermal treatment of HDA-, ODA- and DDA-based perovskites at 100 °C as shown in Figure 3. In agreement with observed PL behavior, both 2D and 1D phases originally present in the ODA sample after 60 min at 100 °C completely decomposed and only diffraction lines belonging to the ODA precursor could been observed (Figure 3a). In the case of the DDA sample, the 1D phase decomposed after 30 min at 100 °C and the XRD pattern showed lines belonging to DDA precursor; however, the 2D DDASnBr_4_ phase still remained present (Figure 3b). Unlike ODA and DDA, thermal treatment of the HDA sample at 100 °C, as shown in Figure 3c, still contained both 2D and 1D phases even after a significantly longer period of 48 h. The rest of the diffraction lines belong to the HDA precursor.

As the HDA sample is significantly more stable compared to the ODA and DDA samples, we have investigated its degradation in more detail by including the additional intermediate temperature as shown in Figure 3d,e. We can observe that the HDA sample after 24 h of thermal treatment at 100 °C, despite the onset of decomposition as evidenced by the presence of HDA precursor diffraction lines, still had a similar ratio of 2D and 1D phases present as it had in the as-prepared sample. Considering that the composition of the ODA and DDA samples shown in Figure 2e both contained dominantly the 1D phase accompanied by small amounts of the 2D phase, it seems reasonable to suspect that the composition of a fresh, as-prepared sample in terms of 2D/1D ratio, in fact, plays a significant role for enhanced stability and prolonged PL of HDA. As both samples (ODA and DDA) with a high ratio of 1D show low stability, we can conclude that the 2D phase is more stable than the 1D phase. However, while DDASnBr_4_ samples after 30 min still contained 2D phase, no emission can be observed without the 1D phase, as the 1D phase is the phase responsible for broad yellow luminescence [7]. The presence of a high proportion of the 2D phase in HDASnBr_4_ likely helps to slow down the degradation of the less stable emissive 1D phase, as it could possibly slow down the ingress of moisture which contributes to the degradation. An additional effect that might contribute to the increased stability of HDA is its crystallinity. We can observe that crystallinity of the ODA and DDA samples is significantly worse compared to HDA sample, as evidenced by the lower signal to background ratio and broadened diffraction lines (Figure 1a–c), indicating that lattice distortions likely increase with the increased length of the spacer cation, resulting in the inferior stability which is in accordance with a previously reported study [3].

**Figure 4:**
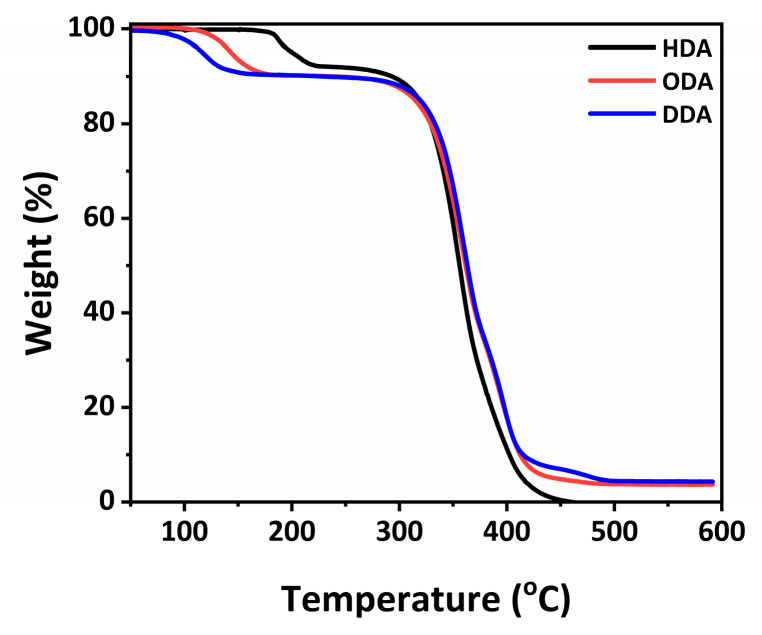
Thermogravimetric analysis plot of ASnBr_4_ perovskites for different spacers A (HDA, ODA, and DDA).

To additionally investigate the degradation of the samples at an elevated temperature, a thermogravimetric analysis (TGA) was performed, as shown in Figure 4. From TGA plots, we can observe that thermal stability in terms of the start of the mass loss trends follows the observations of PL stability with time at 100 °C in ambient air, with the stability increasing with decreasing alkyl chain length HDA > ODA > DDA. Two distinct regions are observed, with the first occurring at temperatures in the range 100–200 °C (exact temperature dependent on the cation used), and the second occurring at a temperature of ~350 °C. For the higher temperature, which likely corresponds to the complete decomposition of the perovskite, the opposite trend is observed with the complete decomposition of HDASnBr_4_ occurring at a lower temperature compared to ODASnBr_4_ and DDASnBr_4_. The samples were stable when heated in an Ar environment, while the emission reduced over the period of two days when heated to 100 °C in ambient air, as shown in Figure S4. A stability comparison at room temperature is shown in Figure S5, and the same trends can be observed as at 100 °C, with HDASnBr_4_ exhibiting the best stability.

It should be noted that in addition to different proportions of the 2D and 1D phases, possible effects of other factors, such as the relationship between organic cation’s carbon chain length and perovskite properties, cannot be entirely excluded. For example, it was previously shown that in quasi-2D Sn-based DJ perovskites, electron and hole diffusion lengths and mobilities were dependent on the spacer cation used [33]; reliable comparisons can only be made for propyldiammonium and butyldiammonium, as other spacer cations resulted in disordered films containing multiple phases. The presence of both the 2D and 1D phases in our samples does not allow us to separate the effect of spacer chemical structure and the effect of the structure (2D:1D phase ratio), as the effects of phase composition dominate the sample properties. We have previously shown that in HDASnBr_4_ there was a very complex relationship between charge carrier dynamics and PL intensity dependence on ambient air exposure [7]. The complexity was attributed to the coexistence of free exciton states in the 2D and 1D phases, where only free excitons of the 1D hydrated phase could form an emissive STE state [7]. Consequently, the sample structure (proportion of 2D and 1D phases and resulting complex excited state dynamics) is expected to dominate the PL observed. However, it is worthwhile to note that, in tin-based perovskites, HDA [3] and hexylammonium (HA) [10] were previously reported to result in the highest PLQY among the different length alkyl chain spacer cations investigated, and HA_2_PbI_4_ exhibited significantly higher carrier mobility compared to both shorter and longer alkyl chains, which was attributed to increased conformational order and lower local chain distortion for HA [34]. As conformational disorder affects the moisture-induced degradation of Pb-based RP perovskites [35], possible effects of spacer cation conformational disorder could be worthwhile to investigate. However, such an investigation should preferably be performed on high quality single crystal samples, rather than powder microcrystals investigated here, and such an investigation is beyond the scope of the work presented here.

**Figure 5:**
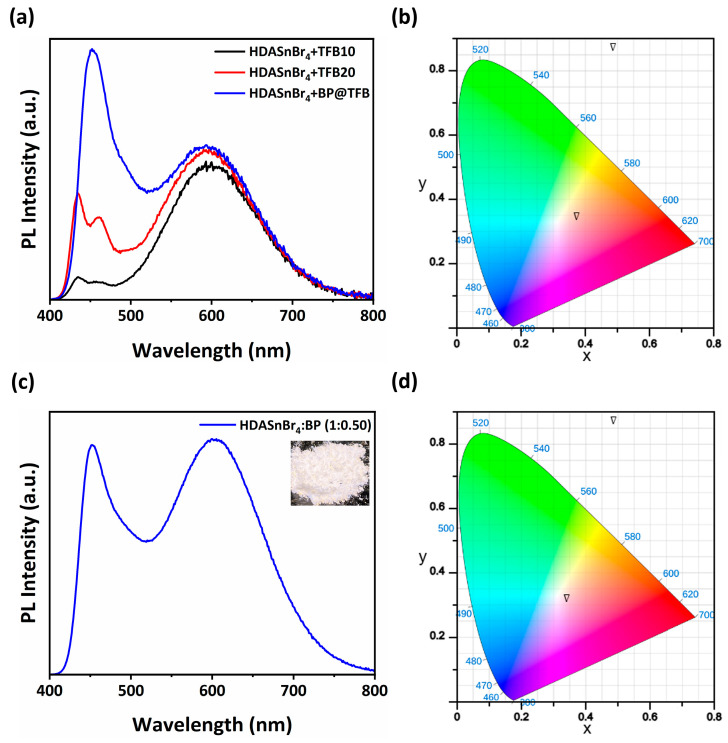
(**a**) PL spectra of HDASnBr_4_ film with different blue-emitting polymer formulations. TFB10 and TFB20 denote 10 and 20 mg of TFB dissolved in 1 mL chlorobenzene, and BP@TFB indicates 10 mg of BP dissolved in TFB solution. (**b**) CIE coordinates chart for HDASnBr_4_+ BP@TFB corresponding to (0.37, 0.34). (**c**) PL spectrum of HDASnBr_4_ BP mixture with a ratio 1:0.50 (inset shows the corresponding photo under UV illumination) and (**d**) corresponding CIE coordinates (0.34, 0.32).

Finally, as HDASnBr_4_ samples exhibit excellent stability, we investigated its application as a phosphor for the achievement of white emission. While powders can be directly used as phosphors, thin films are also of interest due to increased uniformity for large area light sources. However, while prepared powder exhibited strong yellow emission, thin films spin-coated from prepared powder exhibited weaker emission compared to powder; therefore, we explored methods to enhance the emission intensity. It is known that the film stoichiometry can affect the emission efficiency in 2D tin-based perovskites, due to existence of different “dark” and “light” phases [10]. The emission intensity is also affected by the intercalation of water [7] or other solvents, in particular alcohols [21,23,24], and depends on the exact crystal structure of the samples. Thus, we investigated the effect of different solvent vapor on the thin film emission, as shown in Figure S6, and we found a significant enhancement of the light emission in thin films exposed to methanol vapor, in agreement with the expectation that alcohol intercalation increases STE emission [7,21,23]. Thus, warm white emission is obtained when HDASnBr_4_ films are combined with a blue-emitting polymer (poly[(9,9-dioctylfluorenyl-2,7-diyl)-alt-(4,4′-(N-(4-butylphen-yl)))] (TFB)), as shown in Figure 5. The figure also shows the PL spectra and CIE coordinates of the mixed powders of blue phosphor ((Sr,Ba,Ca)_10_(PO_4_)_6_Cl_12_ phosphor, denoted as BP) for the optimal ratio yielding CIE coordinates of (0.34, 0.32). Photos and PL spectra corresponding to other HDASnBr_4_:BP ratios are shown in Figures S7 and S8, respectively.

## 3. Materials and Methods

### 3.1. Experimental Techniques

Ultraviolet absorption (UV-vis) spectra were measured with an Agilent Cary 60 UV-Vis spectrometer (Agilent Technologies, Santa Clara, CA, USA), and photoluminescence (PL) spectra were collected by a PDA-512 USB fiberoptic spectrometer (Control Development Inc., South Bend, IN, USA) with a He-Cd laser (325 nm) as the excitation source. PL spectra of yellow and blue-emitting stacks and mixed powders were measured using a FLS1000 Photoluminescence Spectrometer (Edinburgh Instruments Ltd., Livingston, UK) with excitation at 245 nm, and corresponding CIE color coordinates were extracted with Fluoracle software controlling the spectrometer (Edinburgh Instruments Ltd., Livingston, UK). X-ray diffraction (XRD) patterns were measured by a Rigaku MiniFlex X-ray Diffractometer with CuKα radiation (Rigaku Corporation, Tokyo, Japan). Thermogravimetric analysis (TGA) was performed with a TA Instruments Q50 Thermogravimetric Analyzer (Newcastle, DE, USA) in the temperature range 50–800 °C with a heating rate of 20 °C/min under nitrogen flow at a rate of 60 mL/min.

### 3.2. Synthesis

Materials: Tin(II) bromide (SnBr_2_, >97.0%), dichloromethane anhydrous (DCM, >99%) and hydrobromic acid (HBr, 47%) were purchased from Tokyo Chemical Industry (TCI (Tokyo, Japan)). HDABr_2_, DDABr_2_, and TFB were procured from Xi’an Yuri Solar Co., Ltd (Xi’ian, China). Tetraethyl orthosilicate (TEOS, 99%) was obtained from Dieckmann. N,N-dimethylformamide anhydrous (DMF), dimethyl sulfoxide anhydrous (DMSO), and chlorobenzene anhydrous (CB, 99.8%) were acquired from Alfa Aesar (Haverhill, MA, USA), while Toluene anhydrous (99.8%), methanol (MeOH), ethanol (EtOH), and diethyl ether anhydrous (DE, 99%) were received from Sigma-Aldrich (St. Louis, MO, USA). Blue Phosphor, (Sr,Ba,Ca)_10_(PO_4_)_6_Cl_12_, was obtained from MSE Supplies LLC (Tucson, AZ, USA). All chemicals were used as received without any purification.

Powder Synthesis: An amount of 27.8 mg of SnBr_2_ and organic ammonium spacers (27.8 mg of HDABr_2_, 30.6 mg of ODABr_2_, or 33.4 mg of DDABr_2_) were separately dissolved into pure DMF solvent (0.5 mL) at a fixed ratio of 1:1 in 4 mL glass vials, and then filtered by 0.45 µm polytetrafluoroethylene (PTFE) filters. DCM was slowly diffused into the solution by keeping the above prepared in small vials with lids open in 20 mL capped vials containing 8 mL DCM overnight. In the next step, white powders were collected and washed with DMF and DE three times followed by vacuum-drying overnight. For this experiment, 1, 8-octanediammonium bromide (ODABr_2_) was first synthesized in our laboratory by dissolving 1, 8-octanediamine (440 mg) in ethanol (3 mL) followed by the addition of HBr (780 mL) with continuous stirring, where the ODA to HBr ratio was set to 1:2.2. Next, DE was introduced in an appropriate amount to precipitate the powder. The obtained powder was then collected and redissolved into a mixture of ethanol and water, with further addition of DE for recrystallization; this step was repeated three times to obtain the desired product which was finally vacuum-dried overnight at 60 °C.

Perovskite Film Fabrication: Perovskite precursor solution was prepared by dissolving SnBr_2_ (27.8 mg) and HDABr_2_ (27.8 mg) at a molar ratio of 1:1 in DMF (0.75 mL) and DMSO (0.25 mL) mixed solvent (3:1, *v*/*v*) followed by filtration with 0.45 µm PTFE filter. The concentration of Sn was kept at 0.3 M. Perovskite films were formed on pre-cleaned plasma-treated quartz substrates by spin-coating the precursor solution at 1000 rpm for 5 s and 5000 rpm for 60 s, during which 150 μL of toluene was dripped onto the films as an anti-solvent 35 s prior to the completion of the spin-coating process. Subsequently, the obtained films were annealed on a hot-plate at 90 °C for 10 min.

Film Fabrication for White Emission: To demonstrate white emission, HDASnBr_4_ films were fabricated following the procedure as described above. In order to further enhance yellow emission from the HDASnBr_4_ films, an additional step of solvent vapor annealing was performed using MeOH as the solvent. For this, the pre-developed HDASnBr_4_ films were placed at the center of the MeOH-containing Petri dish covered with a lid for 2 min, after which the films were annealed at 90 °C for 10 min to obtain highly emissive films. For the blue-emitting component, TFB was dissolved in CB in varying concentrations and spin-coated at 2000 rpm for 30 s followed by thermal annealing at 120 °C for 25 min. For achievement of the white emission, yellow and blue films prepared at different concentrations were stacked together.

Powder for White Emission: A total of 50 mg of HDASnBr_4_ powder was taken and blue phosphor was gradually introduced in small quantities with thorough mixing and testing under UV illumination until a white emission was achieved.

## 4. Conclusions

We investigated the stability of different HDASnBr_4_, ODASnBr_4_, and DDASnBr_4_ perovskites heated to 100 °C in ambient air, and we found significantly a higher stability for the HDA samples, where light emission was observed up to 48 h (albeit at a reduced intensity), while negligible emission was obtained in <1 h for ODA and DDA. All the samples consist of a mixture of the 2D and 1D phases, with a proportion of the 2D phase significantly smaller in ODASnBr_4_ and DDASnBr_4_ perovskites, and these samples also have lower crystallinity. The 2D phase degrades slower compared to the 1D phase, as evident from the degradation of the DDASnBr_4_ perovskite, where the 2D phase is still present while the dominant 1D phase has degraded. The improved stability of HDASnBr_4_ can thus be attributed to the higher proportion of a more stable 2D phase, as well as enhanced crystallinity. As the most stable DJ tin-based perovskite investigated, HDASnBr_4_ was then combined with a blue-emitting material to yield white/warm white emission with CIE coordinates of (0.37, 0.34) and (0.34, 0.32) for films and phosphor powder mixture, respectively.

## Data Availability

Data are contained within the article and Supplementary Materials.

## Supplementary Materials

The following supporting information can be downloaded at https://www.mdpi.com/article/10.3390/molecules30030703/s1. Figures S1–S3: PLQY spectra of 50 μm thick layer of powder, several individual microcrystals, and a very low concentration of non-interacting crystals, respectively; Note S1: Discussion of PLQY measurements; Figure S4. PL of HDASnBr_4_ powder as a function of time at 100 °C in Ar and in ambient air; Figure S5. Photos of HDASnBr_4_ films exposed to different solvent vapors; Table S1. Comparison of sample properties for different spacer cations; Figure S6. Photos of samples for different times of exposure to ambient air at room temperature; Figure S7. Photos of phosphors with different ratios of blue phosphor and HDASnBr_4_; Figure S8. PL spectra of phosphors with different ratios of blue phosphor and HDASnBr_4_. Reference [36] is cited in the Supplementary Materials.
