# Supplementary material for: Photoluminescence and Stability of Dion–Jacobson Tin-Based Halide Perovskites with Different Spacer Cation Chain Length
Source: Molecules. 2025 Feb 5;30(3):703. doi: 10.3390/molecules30030703 (PMC11820731; doi:10.3390/molecules30030703)
Supplement: Supplementary file 1 [file molecules-30-00703-s001.zip › molecules-3386682-supplementary.pdf]

SUPPLEMENTARY INFORMATION

Photoluminescence and stability of Dion-Jacobson tin-based halide perovskites with different spacer cation chain length

Muhammad Umair Ali, [1#] Wen Ting Sun,[1#] Aleksandr A. Sergeev,[2] Atta Ur Rehman,[1] Kam Sing Wong,[2] Aleksandra B. Djurišić[1,*] and Jasminka Popović[2,*]

[1] Department of Physics, The University of Hong Kong, Pokfulam Road, Hong Kong

[2] Department of Physics and William Mong Institute of Nano Science and Technology, The Hong Kong University of Science and Technology, Clearwater Bay, Hong Kong

[3]Ruđer Bošković Institute, Bijenička 54, Zagreb, Croatia

#These authors contributed equally.

Note S1. Photoluminescence quantum yield (PLQY) measurements of powder samples can be complex as this type of samples can be susceptible to experimental artefacts, as discussed in the following. Thus, while the general trends between different measurement conditions are consistent (ODA<DDA<HDA), the absolute values shown in Figure **S1** and **S2** cannot be considered as reliable, since the measured value is dependent on the excitation wavelength and the amount of samples, which should not be the case. **We are showing these data to illustrate the common problems with the measurement of PLQY of powder samples**.

PLQY measurements were performed using an Edinburgh Instruments FS-5 spectrometer equipped with the SC-30 integrating sphere module. These types of instruments are very commonly used in the literature. We have initially followed the instructions from manufacturer stating that PLQY from powdered samples is measured using a specific sample holder (a Spectralon dish of about 1 mm depth), which are fully filled with the powdered sample. Non-emissive 5 µm-sized SiO_2 beads were used as a reference to measure the intensity of incident light (so-called, scatter). The measurements were done at 325 nm excitation to match the PL excitation wavelengths used in the study, and PLQY was determined according to the three-measurement procedure [S1]. Surprisingly, this resulted in values exceeding 100% for all three samples (following the trend ODA<DDA<HDA), which are obviously incorrect.

As the most likely explanation for the overestimation of PLQY is the underestimation of the absorbed light by the sample due to the different scattering pattern from reference and target samples (due to different size of scattering centers), we decided to reduce sample thickness, thus reducing the number of scattering and corresponding re-absorption events in the sample. Consequently, a thin (~50 µm) layer of sample powder was mounted between two quartz slides and PLQY was re-measured using two quartz slides as a reference. In addition, excitation wavelength was changed to 400 nm to minimize re-absorption. The obtained results are shown in **Figure S1**, and it can be observed that this approach does not lead to unphysical PLQY values but it results in low signal-to-noise ratio.

To obtain higher signal to noise ratio, we selected excitation wavelength of 380 nm, and we further reduced the sample thickness to only several non-interacting microcrystals to create the thinnest possible layer of powdered sample in the powder holder. In this configuration, the samples' absorption

is very low (see insets in **Figure S2**), resulting in signal intensity for indirect excitation similar to scatter. Consequently, PLQY was determined using only scatter and direct excitation measurements (**Figure S2**). The spectra in **Figure S2** demonstrate that the relative PLQY trend among samples remains consistent, with ODA showing the lowest and HDA showing the highest values, though the difference between them is substantial. Notably, we can confirm that the HDA sample indeed exhibits high PLQY, as evidenced by a well-distinguished PL peak despite very low absorption at the excitation wavelength. These high PLQY values for HDA sample prompted us to consider the possibility of re-absorption events. To investigate this, we conducted an additional series of PLQY measurements using an even lower powder concentration (**Figure S3**). In this case, the PL peak becomes more distinguishable, and the absorption at the excitation wavelength is higher compared to the measurements shown in **Figure S2c**. We attribute this unexpected behavior to the high aggregation tendency of the microcrystals, which results in uncontrollable aggregate formation.

Thus, the obtained results clearly indicate that in this type of samples obtained PLQY values are strongly dependent on the measurement conditions, due to aggregation tendency of microcrystals, re-absorption events, and differences in scattering patterns between microcrystals and reference samples. This makes comparisons of absolute values unreliable, although the relative comparisons (ODA<DDA<HDA) remain consistent. The obtained results also highlight a critical need for establishment of appropriate measurement protocols for aggregating powder samples, as following previously established protocols can lead to overestimated values of >100%.

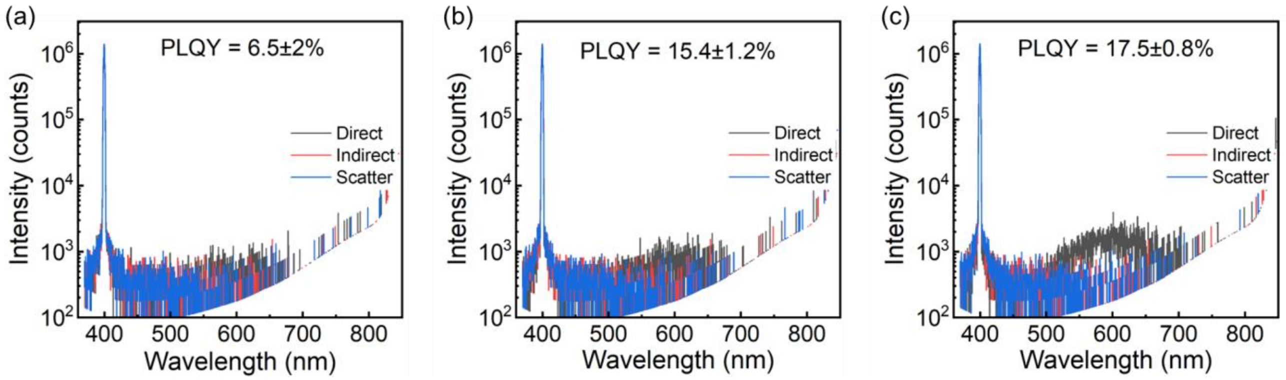

Figure S1. PLQY measurements for ~50 µm-thick powder layer encapsulated between quartz slides. The excitation wavelength is 400 nm. (a) ODA, (b) DDA, (c) HDA.

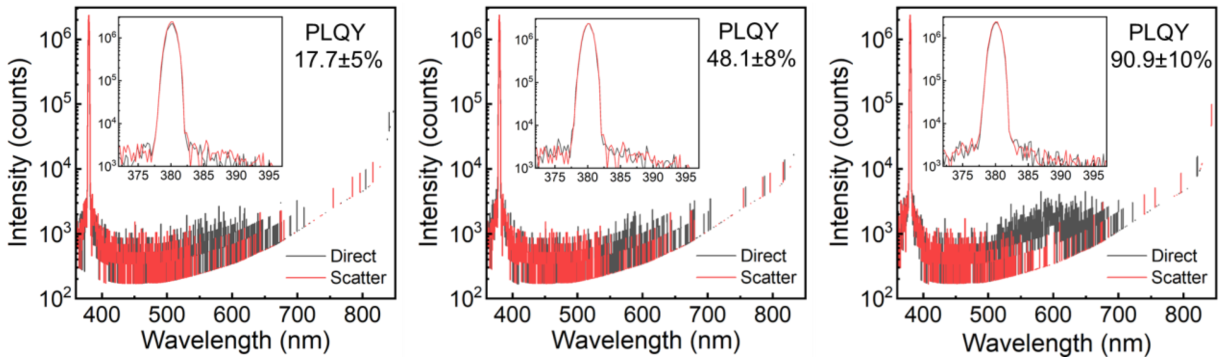

Figure S2. PLQY measurements for several non-interacting microcrystals placed in powder sample holder. The excitation wavelength is 380 nm. (a) ODA, (b) DDA, (c) HDA. Insets show zoomed spectral range for excitation to illustrate the negligible absorption of the samples.

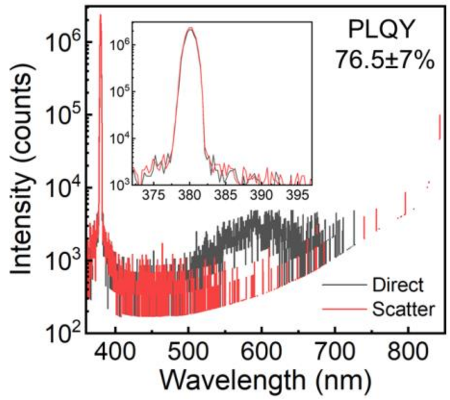

Figure S3. PLQY measurements of HDA sample at very low concentration of non-interacting microcrystals placed in powder sample holder.

Table S1. Comparison of sample properties for different spacer cations.

Sample	1D structure /wt. %	2D structure /wt. %
HDA	33(1)	67(2)
ODA	79(1)	21(2)
DDA	81(2)	19(1)

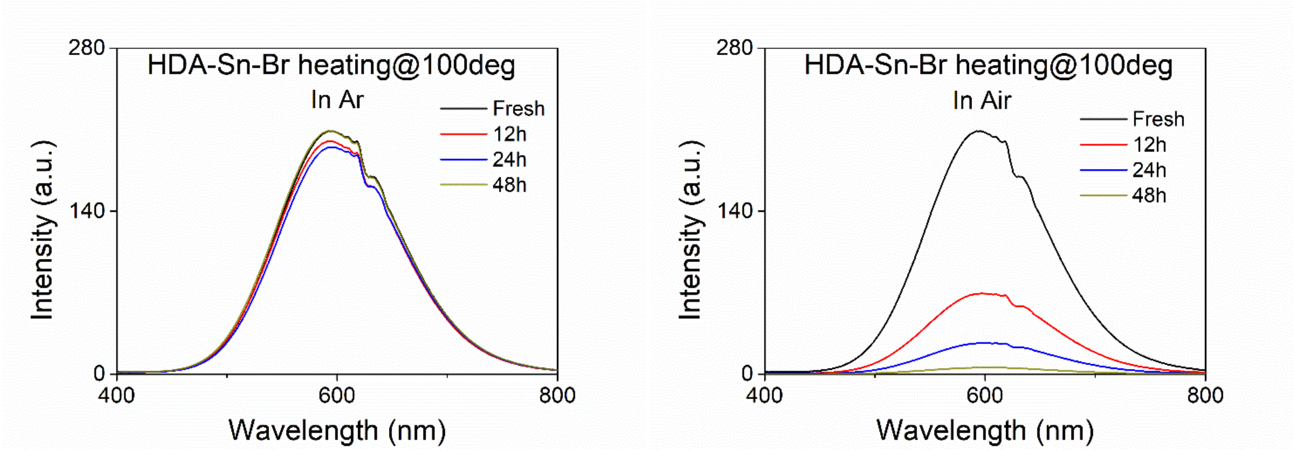

Figure S4. PL spectra of $HDASnBr_4$ as a function of time at 100ºC in a) Ar b) ambient air.

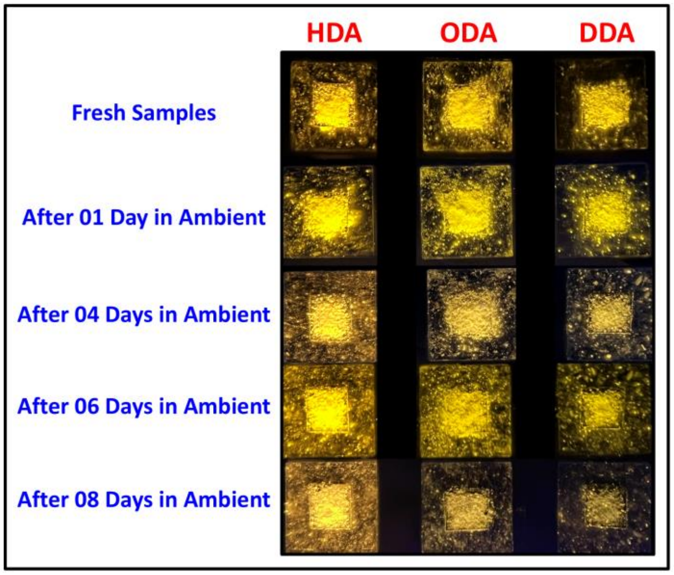

Figure S5. Photos of $HDASnBr_4$, $ODASnBr_4$ and $DDASnBr_4$ under UV illumination for different times of exposure to ambient air (RH: ~40-45%) at room temperature.

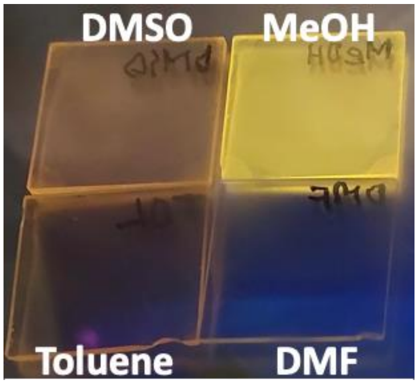

Figure S6. Photos of $HDASnBr_4$ films under UV illumination at 254 nm exposed to different solvent vapor.

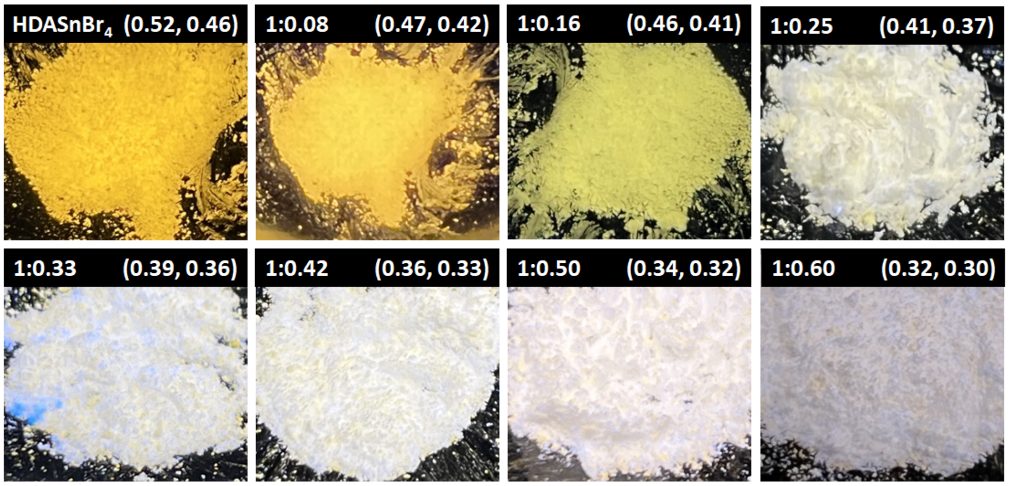

Figure S7. Photos of phosphors with different ratios of blue phosphor and $HDASnBr_4$ under ultraviolet (UV, 365 nm) illumination. The sample labels are shown on the top left corners and corresponding CIE coordinates are specified on the top right corners.

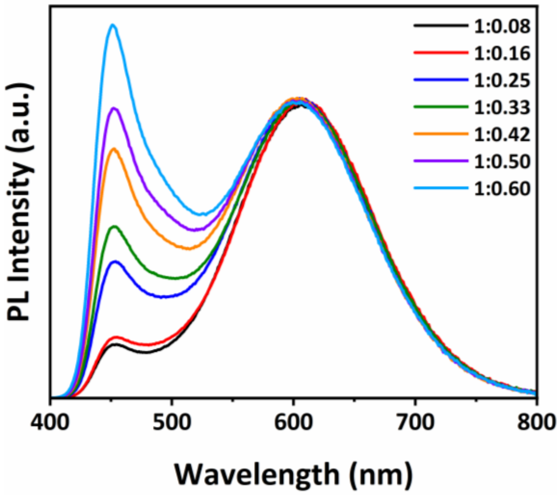

Figure S8. PL spectra of phosphors with different ratios of blue phosphor and $HDASnBr_4$.

References:

S1. de Mello, J. C.; Wittmann, H. F.; Friend, R. H. An improved experimental determination of external photoluminescence quantum efficiency. *Adv. Mater.* **1997**, *3*, 230-232.
